# The Peptide Chain Release Factor Methyltransferase PrmC Influences the *Pseudomonas aeruginosa* PA14 Endo- and Exometabolome

**DOI:** 10.3390/metabo10100417

**Published:** 2020-10-18

**Authors:** Tobias Depke, Susanne Häussler, Mark Brönstrup

**Affiliations:** 1 Department of Chemical Biology, Helmholtz Centre for Infection Research, 38124 Braunschweig, Germany; tobias.depke@helmholtz-hzi.de; 2 Institute of Molecular Bacteriology, Twincore, Centre for Clinical and Experimental Infection Research, 30625 Hannover, Germany; 3 Department of Molecular Bacteriology, Helmholtz Centre for Infection Research, 38124 Braunschweig, Germany; 4 German Centre for Infection Research (DZIF), Partner Site Hannover-Braunschweig, 38124 Braunschweig, Germany

**Keywords:** *Pseudomonas aeruginosa*, PrmC, metabolomics, virulence

## Abstract

*Pseudomonas aeruginosa* is one of the most important nosocomial pathogens and understanding its virulence is the key to effective control of *P. aeruginosa* infections. The regulatory network governing virulence factor production in *P. aeruginosa* is exceptionally complex. Previous studies have shown that the peptide chain release factor methyltransferase PrmC plays an important role in bacterial pathogenicity. Yet, the underlying molecular mechanism is incompletely understood. In this study, we used untargeted liquid and gas chromatography coupled to mass spectrometry to characterise the metabolome of a *prmC* defective *P. aeruginosa* PA14 strain in comparison with the corresponding strain complemented with *prmC in trans*. The comprehensive metabolomics data provided new insight into the influence of *prmC* on virulence and metabolism. *prmC* deficiency had broad effects on the endo- and exometabolome of *P. aeruginosa* PA14, with a marked decrease of the levels of aromatic compounds accompanied by reduced precursor supply from the shikimate pathway. Furthermore, a pronounced decrease of phenazine production was observed as well as lower abundance of alkylquinolones. Unexpectedly, the metabolomics data showed no *prmC*-dependent effect on rhamnolipid production and an increase in pyochelin levels. A putative virulence biomarker identified in a previous study was significantly less abundant in the *prmC* deficient strain.

## 1. Introduction

The ubiquitous γ-proteobacterium *Pseudomonas aeruginosa* is an important causative agent of infections in predisposed individuals such as those suffering from cystic fibrosis or burn wounds [1]. Deemed a priority antibiotic resistant bacterium by the World Health Organisation (WHO) [2], it poses a considerable threat to public health, and causes a high number of nosocomial infections [3,4]. *P. aeruginosa* possesses an arsenal of virulence factors that is intricately regulated by quorum sensing systems and activated in response to environmental factors via multiple signalling pathways [5]. While a large number of proteins and pathways have known roles in the regulation of virulence, there are enzymes that contribute to a virulent phenotype by an unknown mechanism. The *S*-adenosyl-L-methionine-dependent peptide chain release factor methyltransferase PrmC (synonym: HemK) belongs to the latter group. Its absence results in a reduction of in vivo virulence—specifically the inability to produce pyocyanin—and an impaired adaptation to challenging environments [6]. Its original role is to posttranslationally methylate the release factors RF-1 and RF-2 (PrfA and PrfB in *P. aeruginosa*), thereby facilitating translation termination and decreasing the rate of stop-codon readthrough [7,8]. Previous transcriptomic and proteomic analyses gave new insight and suggested that PrmC activity alters mRNA-protein ratios depending on which stop codon is used [6,9]. In this study, we have applied metabolomics, the characterisation of the set of small-molecule primary and secondary metabolites, as a complementary technology to capture the effects of *prmC* deficiency in *Pseudomonas aeruginosa* PA14. The exometabolome, i.e., the metabolites secreted into and consumed from the growth media, was recorded along with the cellular metabolome, i.e., the endometabolome. Furthermore, liquid chromatography–mass spectrometry (LC–MS) and gas chromatography–mass spectrometry (GC–MS) were combined for advanced analytical coverage.

## 2. Materials and Methods

To ensure comparability to previous research, the same bacterial strains as in Krueger et al. were used [9]: The *P. aeruginosa* PA14 *prmC* transposon mutant from the Harvard PA14 mutant library [10] carrying an empty pUCP20 vector, abbreviated PA14 tn*prmC* and the same strain complemented with the *prmC* gene on the vector, PA14 tn*prmC*::*prmC*. In order to assure that all phenotypic effects are due to the absence or presence of the *prmC* gene, and to exclude that effects are caused by secondary mutations that might occur in addition to the inactivation of *prmC* in the transposon mutant, the transposon mutant was not compared to the corresponding wildtype, but to an equivalent, *prmC*-complemented transposon mutant. Five biological replicates of each strain were cultivated in glass flasks in 60 mL modified BM2 medium supplemented with additional amino acids (7 mM (NH_4_)_2_SO_4_, 40 mM K_2_HPO_4_, 22 mM KH_2_PO_4_, 2 mM MgSO_4_, 10 µM FeSO_4_ with 0.01% casamino acids) in a shaking incubator (approx. 150 rpm) at 37 ${}^{{}^{\circ}}$C . Blank samples (pure media without inoculation) were prepared accordingly and processed like the bacterial samples throughout the experiment. Bacteria were grown until an OD_600_ of 1.6–1.7 before harvesting. Each biological replicate was split into two technical replicates for GC–MS analysis and two for LC–MS analysis by transferring 20 mL and 5 mL, respectively, to pre-cooled tubes, which were then immediately centrifuged at 9000× *g* for 10 min at 4 ${}^{{}^{\circ}}$C. 1.5 mL supernatant was transferred from the LC–MS samples to fresh tubes and dried overnight in a centrifugal evaporator at 20 ${}^{{}^{\circ}}$C and full vacuum until complete dryness as described before [11]. The cell pellets for both LC–MS and GC–MS samples were snap frozen in liquid N_2_. Cell pellets for GC–MS analysis were resuspended in 1.5 mL 75% (*v*/*v*) methanol with 2 mg/L ribitol as an internal standard and supplemented with 1.5 mL deionised water and chloroform each, followed by vigorous shaking. The organic phase was discarded, and 1 mL of the aqueous phase was evaporated to dryness. Sample preparation, GC–MS analysis, data pre-processing and metabolite identification were performed as described by Berndt et al. [12]: Metabolite extracts were derivatised by methoxyamine/pyridine and silylated using MSTFA to volatilise the analytes. The samples were separated on a 30 m column with a stationary phase of 5% phenyl arylen and 95% dimethylpolysiloxane material (ZB-5MS^®^, Phenomenex, Torrance, CA, USA) using a temperature gradient from 70 ${}^{{}^{\circ}}$C to 330 ${}^{{}^{\circ}}$C over 32.5 min. MS data was recorded in full scan mode by electron impact ionisation in positive mode on an iontrap mass spectrometer (Thermo Scientific ITQ 900™, Thermo Fisher Scientific, Waltham, MA, USA). A mix of alkanes (chain length 10 to 36) was used to calibrate the retention index of the analytes. Spectral deconvolution, alignment and annotation were achieved using the MetaboliteDetector software [13] and an in-house spectral library. Directional fold changes were calculated by comparison of the mean feature intensities of the two strains and *p*-values were calculated using Welch’s *t*-test with Benjamini–Hochberg correction for multiple testing. Four replicates (two of tn*prmC* samples and two of tn*prmC*::*prmC* samples) were lost for data analysis due to a technical problem with the GC–MS instrument. Cell pellets and dried supernatants for LC–MS analysis were extracted using 1 mL and 500 μL 100% methanol containing 0.1 mg/L trimethoprim, 0.1 mg/L nortriptyline and 0.3 mg/L glipizide as internal standards, respectively, by vigorous shaking, followed by sonication in a cooled ultrasonic bath for 15 min. After centrifugation, 800 μL and 400 μL, respectively, were transferred to fresh tubes, evaporated to dryness and reconstituted in 80 μL/40 μL 50% acetonitrile containing 1 mg/L caffeine and 8 mg/L naproxen as internal standards, respectively, sonicated and centrifuged again and then used as LC–MS samples. Pooled samples containing aliquots of all samples were generated for quality control purposes. LC–MS analysis, pre-processing using XCMSonline [14], metabolite identification and statistical analysis were performed as described previously [11,15]. In brief, the metabolite extracts were separated by ultra-high perfomance liquid chromatography on a C18 reversed phase column by gradient elution with H_2_O plus 0.1% formic acid and acetonitrile plus 0.1% formic acid. The MS data was recorded by a quadrupole time-of-flight mass spectrometer (maXis™ HD QTOF, Bruker, Bremen, Germany) after electrospray ionisation in positive mode. Pooled samples were additionally subjected to collision-induced dissociation by Bruker’s data-dependent auto-MS/MS functionality to record tandem mass spectra for metabolite identification by comparison to authentic chemical standards and/or metabolite databases. Data pre-processing by XCMS used the same parameters as an earlier study [15]. Further processing involved exclusion of features eluting in the first 0.8 min or after more than 20 min, features with a standard deviation below 20% over all samples and features with an intesity below 10,000 counts. The data was then normalised on the levels of the internal standards and differences in the amount of biomaterial was accounted for by normalising on the OD_600_ of the respective samples. Isotopes and adducts identified by CAMERA [16] were also excluded. Annotations were added and the resulting feature tables were used for all analyses. Directional fold changes were and *p*-values were calculated as described for the GC–MS data. Throughout data processing, intra- and inter-group variability were assessed with the help of principal component analysis and by monitoring the levels of internal standards.

## 3. Results and Discussion

**Figure 1:**
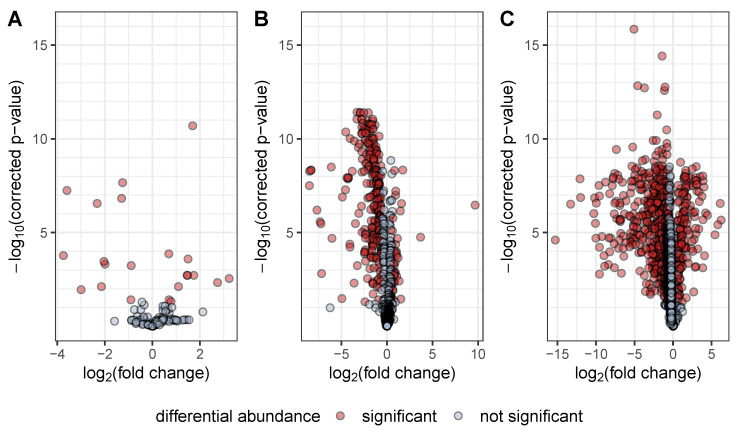
Volcano plots of GC–MS metabolomics data (**A**), LC–MS endometabolomics data (**B**) and LC–MS exometabolomics data (**C**). All features of the respective data sets are plotted by their log_2_(fold change) and ‒log_10_(corrected *p*-value). Features are considered significantly differentially abundant if their fold change exceeds 1.5 or ‒1.5 and their corrected *p*-value is at most 0.05. While similar numbers of features displayed higher and lower abundance, respectively, in the GC–MS data, both endo- and exometabolomics LC–MS data showed a higher proportion of significantly decreased feature intensities, highlighting the dampening effect of PrmC deficiency on the production of secondary metabolites. For the GC–MS data, only identified features and reproducible unknowns were analysed, explaining the difference in overall feature number to the LC–MS data. Two features which were not detected in tn*prmC*, shikimate and shikimate-3-phosphate, are missing in the GC–MS volcano plot since no fold changes could be calculated.

*P. aeruginosa* PA14 with and without a functional *prmC* gene showed various differences in their metabolic profiles (Figure 1). Differences were less pronounced in the part of the metabolome accessible to GC–MS analysis. 24 of 116 features were significantly differentially abundant with a directional fold change ≥1.5 or ≤‒1.5 and a Benjamini–Hochberg corrected *p*-value ≤ 0.05. However, a substantial proportion of the endo- and exometabolome measured by LC–MS was affected by the absence of *prmC*. 291 of 763 features and 893 of 1780 features were significantly differentially abundant, respectively. In both LC–MS data sets, decreased abundance upon loss of *prmC* dominated over increased abundance (Figure 1), which is in concordance with previous research showing that more proteins and transcripts were down-regulated than up-regulated in *prmC* deficient strains [9].

The GC–MS metabolomics data, that generally represent primary and intermediary metabolism, showed few consistent trends for metabolic pathways affected by *prmC* deficiency. Nevertheless, a consistent finding was that aromatic compounds were depleted in strains lacking *prmC*. For example, phenylalanine and tyrosine were significantly less abundant, with directional fold changes of ‒2.5 and ‒4.4, respectively (Supplementary Table S1). Strikingly, shikimate and shikimate-3-phosphate, biogenic precursors of most phenylic compounds, could not be detected in any of the tn*prmC* samples, whereas they are present in tn*prmC*::*prmC*. This could point towards a general down-regulation of the shikimate pathway with downstream effects on secondary metabolite production. Specifically, this seems to affect alkyl quinolones and phenazines whose production relies on precursors derived from shikimate [17,18]. Although other intermediates from the pathway were not detected, 3-deoxyarabinoheptulosonate was found to be 1.8 times less abundant in tn*prmC*. This feature could be a fragment or degradation product of 3-deoxy-arabino-heptulosonate 7-phosphate (DAHP), a precursor of shikimate. In the proteomic data of Krueger et al., the only enzyme involved in the shikimate pathway with a significant difference between tn*prmC* and tn*prmC*::*prmC* was 3-phosphoshikimate-1-carboxyvinyl-transferase (PA14_23310) [9]. This enzyme acts downstream of shikimate-3-phosphate and thus its downregulation cannot directly explain shikimate and shikimate-3-phosphate depletion. On the mRNA level, the transcripts for *phzC1* and *phzC2* were significantly downregulated in tn*prmC* [9]. The gene product PhzC is a DAHP synthase that complements other DAHP synthases such as AroF and is believed to control flux of precursors into phenazine biosynthesis [18].

Depletion of aromatic compounds (i.a., phenylalanine, tyrosine, anthranilate) in the absence of *prmC* was also observed in the LC–MS endo- and exometabolomics data (Supplementary Tables S2 and S3). From their lower abundance in the spent media, it might be concluded that reduced activity of the shikimate pathway is counteracted by an increased uptake of aromatic amino acids from the medium, thereby ensuring a near-normal growth phenotype.

**Figure 2:**
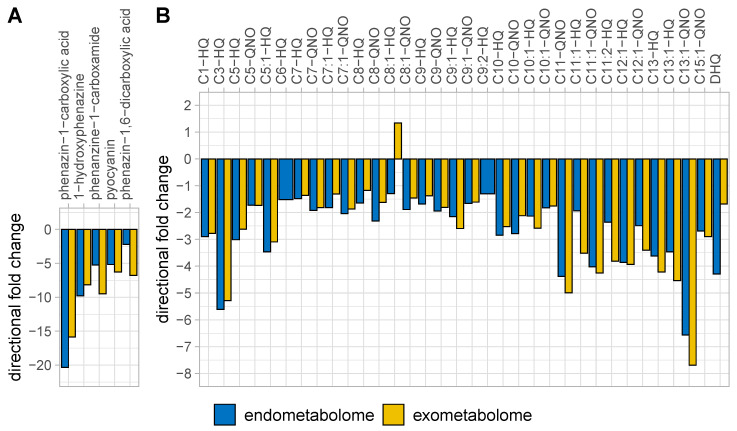
Directional fold changes of phenazines (**A**) and AQs (**B**) in the LC–MS endo- and exometabolomics data. All detected phenazines were much less abundant in tn*prmC* with no clear difference between cellular and extracellular levels. AQs were consistently less abundant in tn*prmC*. This trend was more pronounced for AQs with very long or very short alkyl chains. Panel A is sorted by magnitude of directional fold change, panel B by the length of the alkyl chain of the respective AQ. Panel B uses the nomenclature of Depke et al. [11]. While technically not an AQ, 2,4-dihydroxyquinoline (DHQ) has been added as a by-product of AQ biosynthesis. Only significantly differentially abundant features (corrected *p*-value ≤ 0.05) are shown. C6-HQ and C9:2-HQ were not significantly differentially abundant in the exometabolome.

The metabolomic effects of *prmC* deficiency were strongest on phenazine and alkylquinolone (AQ) levels, which was expected from previous studies [6,9]. It was found that not only pyocyanin, but all phenazines, were consistently less abundant both in the cellular metabolome and in the exometabolome, if *prmC* was non-functional (Figure 2A). The same was true for AQs but to a lesser degree (Figure 2B). Consistent with this phenomenon and the generally lower levels of aromatic metabolites, LC–MS endometabolomics also showed decreased levels of anthranilate in tn*prmC* (directional fold change ‒2.0, corrected *p*-value 3.4 × 10^‒8^), a direct precursor of alkyl quinolones. The levels of the short or especially long alkyl chain AQs appeared to be more strongly affected by *prmC* deficiency, whereas the effect on the medium chain lengths (C6 – C9) was less pronounced. This is in accordance with recent findings on the substrate specificity of the biosynthetic enzyme complex PqsBC, which prefers medium chain activated fatty acids over long and short chain ones [19]. Krueger et al. demonstrated lower levels of PqsB and PqsC in tn*prmC* [9]. Thus, the reduced abundance of PqsBC might lead to higher relative consumption of the preferred substrates, while the less favoured long and short chain substrates are neglected.

Rhamnolipids are other important components of pseudomonal small molecule mediated virulence. Members of this class of metabolites were not consistently affected by *prmC* deficiency. In the extracellular metabolome, none of the features that were annotated as a rhamnolipid met the significance criteria (directional fold change ≥ 1.5 or ≤ ‒1.5 and corrected *p*-value ≤ 0.05). In the endometabolome, only one of the rhamnolipids was significantly differentially abundant. This feature was more abundant in tn*prmC*. These findings contradict those of Pustelny et al., who found reduced rhamnolipid levels in tn*prmC* [6]. The discrepancy might be explained by the later growth phases at the time of measurement: Pustelny et al. quantified rhamnolipids after 48 h of growth. The effect of *prmC* on rhamnolipid production might be time and/or growth phase-dependent and might not manifest before stationary phase.

Interestingly, a significantly higher abundance of the two pyochelin isomers was detected in tn*prmC* (fold changes +2.8 and +1.9 in the endometabolome and +2.6 and +1.8 in the exometabolome; all corrected *p*-values < 10^‒4^). Pyochelins are low affinity siderophores of *P. aeruginosa*, whose production is coordinated with pyoverdine, a high affinity siderophore [20]. Unfortunately, pyoverdine was not detected by our analytical methods, which complicates the interpretation of elevated pyochelin levels. Earlier research has associated elevated pyochelin levels with a virulent phenotype [15,21,22], and proteome and transcriptome data show the *pchD* transcript as being upregulated in tn*prmC* [9].

Among the non-annotated features, it is particularly noteworthy that M187T7, a metabolite with an *m/z* of 187.1230 and the putative sum formula C_12_H_15_N_2_, displays a significantly differential abundance with approximately 5-fold lower levels in tn*prmC* and a corrected *p*-value of 0.0001 in the cellular metabolomics data (fold change ‒2.8 and corrected *p*-value 0.016 in the exometabolome). The ‘unknown’ metabolite has been identified as a putative biomarker for virulent phenotypes in clinical *P. aeruginosa* strains in a previous study [15]. Though its identity and function are unassigned, this finding emphasises the potential importance of the metabolite for pseudomonal virulence.

There is a substantial number of additional non-annotated features that show highly significant differences in abundances between tn*prmC* and tn*prmC::prmC*, suggesting an influence of *prmC* on other parts of pseudomonal metabolism. While the annotated significantly affected metabolites in the endometabolome measured by LC–MS are almost exclusively directly or indirectly associated with virulence as described above, the respective list for the exometabolome also comprises glutathion disulphide with a directional fold change of +7.5 and a corrected *p*-value of 1.2 × 10^‒8^. This indicates further consequences of *prmC* deficiency on the redox regulation of *P. aeruginosa*, which could potentially be linked to the distorted phenazine production. In the GC–MS metabolomics data, some features stand out by their low *p*-value and/or prominent fold change, but their connections to *prmC* cannot be easily established. For instance, succinate is depleted in *prmC* deficient bacteria (fold change –2.4, corrected *p*-value 2.2 ×10^‒8^), whereas acetylserine is significantly more abundant in tn*prmC* (fold change +9.4, corrected *p*-value 0.0028). These findings show that the complexity of the metabolic consequences of *prmC* deficiency is considerable and far from being completely understood.

While this study consists of a comprehensive metabolomic profiling experiment, it also comes with several limitations. Like many untargeted metabolomics investigations, the data presented here suffers from incomplete metabolite annotation that leaves several interesting pathways underexplored. Furthermore, metabolic fluxes have not been determined which hinders quantitative correlation with transcriptome data. Along with the novel findings outlined above, most results obtained by means of untargeted metabolomics are consistent with previous transcriptomic and proteomic studies [6,9] and support and complement their findings.

## 4. Conclusions

In conclusion, we present the first metabolomics study of the role of *prmC* in virulence and metabolism of *P. aeruginosa*. The results support findings from earlier and complementary -omics studies [6,9]. In addition, they shed light on the importance of the shikimate pathway on important AQ and phenazine virulence-mediating metabolites at high molecular resolution and highlight the potential of the putative novel virulence marker M187T7.

## Supplementary Materials

The following are available online at https://www.mdpi.com/2218-1989/10/10/417/s1, Table S1: Feature table of the GC–MS data, Table S2: Feature table of all annotated features of the LC–MS endometabolomics data, Table S3: Feature table of all annotated features of the LC–MS exometabolomics data. Metabolomics data have been deposited to the EMBL-EBI MetaboLights database (DOI: 10.1093/nar/gkz1019, PMID:31691833) with the identifier MTBLS2046. The complete dataset can be accessed here https://www.ebi.ac.uk/metabolights/MTBLS2046.

## Abbreviations

The following abbreviations are used in this manuscript:

WHO	World Health Organisation
RF	release factor
LC–MS	liquid chromatography–mass spectrometry
GC–MS	gas chromatography–mass spectrometry
MSTFA	*N*-methyl-*N*-(trimethylsilyl)trifluoracetamide
DAHP	3-deoxy-arabino-heptulosonate 7-phosphate
AQ	alkylquinolone
DHQ	2,4-dihydroxyquinoline
C*n*:*m*	hydrocarbon of chain length *n* with *m* double bonds
HQ	hydroxyquinoline
QNO	quinolone-*N*-oxide
